# The Wnt antagonist and secreted frizzled-related protein 5: implications on lipid metabolism, inflammation, and type 2 diabetes mellitus

**DOI:** 10.1042/BSR20180011

**Published:** 2018-07-03

**Authors:** Ling-Bin Liu, Xiao-Dong Chen, Xiang-Yu Zhou, Qing Zhu

**Affiliations:** 1 Farm Animal Genetic Resources Exploration and Innovation Key Laboratory of Sichuan Province, Sichuan Agricultural University, Chengdu 611130, China; 2 Department of Thyroid Surgery, The Affiliated Hospital of Southwest Medical University, Luzhou 646000, China

**Keywords:** Inflammation, Lipid metabolism, Secreted frizzled-related protein 5, Type 2 diabetes mellitus, Wnt signaling pathway

## Abstract

Various reports have suggested that secreted frizzled-related protein (SFRP) 5 (SFRP5) plays a regulatory role in the processes of cellular proliferation and differentiation, by means of inactivating the Wnt/β-catenin signaling pathway. Recently, SFRP5 has been identified as an anti-inflammatory adipokine, which may be induced during preadipocyte proliferation, differentiation, and maturation. This review aims to identify the recent progress in the research and development of SFRP5 that can play a role in influencing lipid metabolism, inflammation, and type 2 diabetes mellitus (T2DM). Recent evidence has indicated that SFRP5 is capable of stimulating adipocyte differentiation via inhibition of the Wnt/β-catenin signaling pathway. In addition, SFRP5 binding with wingless-type murine mammary tumor virus integration site family, member 5A (Wnt5a), inhibits the activation of c-Jun N-terminal kinase (JNK) downstream of the Wnt signaling pathway. An antagonistic relationship has been found between the reductions in inflammatory cytokine production and serine phosphorylation of insulin receptor substrate-1 (IRS-1) in regard to inhibition of insulin signaling network. By this mechanism, SFRP5 exerts its influence on metabolic function. Based on our review of the current available literature, we support the notion that SFRP5 can be used as a therapeutic target in the treatment of T2DM.

## Introduction

Secreted frizzled-related proteins (SFRPs) consist of five identified secreted glycoproteins, which have been identified as negative modulators of the Wnt signal transduction pathway [1,2]. SFRP5, is a member of the SFRP family produced from adipose tissue and can act as an inflammatory adipokine. SFRP5 has been shown to act as an antagonist for the Wnt/β-catenin signaling pathway [3]. It has recently gained much attention, owing to the notion that it is capable of promoting adipocyte differentiation via blockade of the Wnt/β-catenin signaling pathway, thus leading to an increased risk of diet-induced obesity [4,5]. Initially, Chang et al. [6] found that the human *SFRP5* gene is composed of three coding exons, all of which are located on chromosome 10q24.1. SFRP5 has been reported to exert its effects by regulating the Wnt signal transduction, by competing with membrane frizzled receptors that function as a binding domain for secreted Wnt ligands [7].

As functional antagonists, both the Wnt family and SFRPs control multiple biological processes, including that of embryonic development, inflammation, and immunity [8]. SFRPs block the Wnt signaling pathway either by interacting with Wnt proteins and preventing them from binding to their frizzled target proteins, which are receptors that act as positive mediators of the Wnt signaling pathway or by forming nonfunctional complexes with frizzled proteins [9,10]. The SFRP family molecules contain a netrin-like functional domain as well as a cysteine-rich domain both of which exhibit a close homology with the frizzled cysteine-rich domain, allowing them to outcompete the frizzled protein and suppress the Wnt signaling pathway [11]. Additionally, SFRPs compete to bind to Wnt ligands and inhibit signal transduction via frizzled receptors through the cysteine-rich domains [12]. Christodoulides et al. [13] reported that SFRP5-mediated epigenetic silencing of the Wnt signaling pathway in white adipose tissues could lead to strengthened adipogenesis with a significant likelihood increasing susceptibility to diet-induced obesity in mice models. In addition, SFRP5 has been demonstrated to inhibit the activation of c-Jun N-terminal kinase (JNK) downstream of the Wnt signaling pathway [14,15]. The antagonism of serine phosphorylation by SFRP5 on the insulin receptor substrate-1 (IRS-1) has been shown to inhibit the insulin signaling network. SFRP5 suppresses chronic inflammation and can consequently improve insulin sensitivity, highlighting its role as an independent risk factor for type 2 diabetes mellitus (T2DM), with a notably important role in the maintenance of glucose homeostasis [16]. Thus, the primary purpose of this article was to review the underlying mechanisms by which SFRP5 mediates lipid retention in adipocytes while highlighting its relationship with the mechanisms of lipid metabolism, inflammation, and T2DM.

## The concept of the Wnt antagonist SFRP5

Wnt signaling has been categorized into three distinct pathways: the canonical Wnt signaling pathway involving β-catenin signaling, the non-canonical planar cell polarity pathway, and the Wnt and calcium pathway [17]. The Wnt family proteins activate signaling via binding to one or more frizzled family receptors [18]. As functional antagonists, the wingless/integrase-1 (Wnt) family and SFRPs regulate a variety of biological processes, including embryonic development, inflammation, and immunity. When there is a lack of Wnt antagonizing signals, the canonical Wnt signaling pathway is activated, which leads to stabilization and hypophosphorylation of β-catenin. The hypophosphorylated β-catenin then goes on to interact with transcription factors in the T cell factor family after translocation in the nucleus [19]. It has been found that SFRP5 inhibits both canonical and non-canonical Wnt signaling pathways during embryonic and endodermal development [20]. Li et al. [21] demonstrated that SFRP5 antagonizes both canonical and non-canonical Wnt11 signaling, showing that SFRP5 binds to and competes for Wnt5 and Wnt11 proteins. The successful competitive inhibition of the endogenous Wnt11 signaling protein results in the activation of both the canonical and non-canonical pathways [21]. Wnt proteins expressed or secreted from the surface of the signaling cells exert their downstream effects by binding to the frizzled and low-density lipoprotein (LDL) receptor-related protein (LRP) complex. These receptors transduce signals to intracellular proteins, including Disheveled (Dvl), the scaffold protein Axin, glycogen synthase kinase-3β (GSK-3β), adenomatous polyposis coli (APC), as well as β-catenin, which acts as a transcriptional regulator [9]. The expression levels of β-catenin in the cytoplasm are generally regulated by proteasome degradation in order to maintain its concentration at relatively low levels [10]. β-catenin degradation is chiefly regulated by the GSK-3/APC/Axin complex within the cytosol. The β-catenin degradation is averted when cells respond to Wnt signals, leading to an increase in β-catenin accumulation within the nucleus and cytoplasm. Nuclear β-catenin interacts with transcription factors including that of lymphoid enhancer-binding factor 1/T-cell-specific transcription factor (LEF-1/TCF), which subsequently influences gene transcription of downstream genes, such as *CCND1, c-myc, Axin2, and MMP-7* [22]. Reports have revealed that SFRP5 initially binds with Wnt5a to prevent JNK activation in the downstream of Wnt signaling pathway in adipose tissues. As a result, we discovered a subsequent decrease in the production of pro-inflammatory cytokines, and antagonization of the IRS-1 Ser^307^ phosphorylation [23]. Thus, SFRP5 has the ability to modulate the progression of T2DM as a result of its insulin-sensitizing and anti-inflammatory properties which have all been well documented [24]. Anti-inflammatory and antidiabetic effects have been observed with regard to the antagonism of Wnt5a by SFRP5 through the Wnt signaling pathway [25]. SFRP5 not only strengthens adipocyte hypertrophy through repressing oxidative metabolism, but also serves as an anti-inflammatory adipokine, which modulates both inflammation and metabolic dysfunction [26]. Understanding the underlying mechanisms of how SFRP5 can modulate adipogenesis and energy homeostasis highlights its critical role in combating significant public health issues in the form of obesity and T2DM.

## SFRP5 and adipogenesis

SFRP5 has been found to help increase lipid accumulation in adipocytes in order to allow adipose tissue expansion to occur. These effects are a result of increased mitochondrial respiration elicited by SFRP5 suppressive effects on oxidative metabolism by blocking Wnt signaling by means of preventing the binding of Wnt proteins to their respective receptors [27]. The role of Wnt signaling, as an adipogenesis switch, in blocking the Wnt ligands which inhibit adipogenesis and adipocyte lipid accumulation has been elucidated [28]. A number of hypotheses have been put forward suggesting that the SFRP5-Wnt5a modulatory axis in adipose tissues presents a novel promising target for controlling metabolic disorders [29]. Wnt5a consists of a key ligand and antagonist pair with SFRP5 [8]. Wnt5a is produced by macrophages in adipose tissues, which in turn acts to influence adipocyte function, while Wnt5a signaling, in chronic low-grade inflammation, is balanced by its inhibitor, SFRP5 [14]. Wnt proteins repress adipocyte differentiation by decreasing the expression levels of pre-adipogenic transcription factors including members of the C/EBP family and peroxisome proliferator-activated receptor γ (PPARγ) [30]. The PPARγ nuclear receptor regulates the initial stages of preadipocyte differentiation into lipid accumulating adipocytes as well as repressing Wnt signaling by enhancing proteasome-dependent β-catenin degradation [31]. Blockade of Wnt signaling by anti-inflammatory SFRP5 is a key process involved in the initiation of adipogenic differentiation [4,14]. The action of the Wnt/β-catenin is suppressed by three main factors: extracellularly produced SFRPs, which dissociate Wnt ligands; extracellularly produced Dickkopf, which blocks LRP coreceptors; and intracellularly produced Dapper, which binds to Disheveled (Dvl) [32]. A wide consensus exists suggesting that Wnt10b acts as a positive mediator of Wnt signaling and exerts its effects on adipocyte development by reducing the expression levels of PPARγ and C/EBPα [33,34]. The binding of the positive modulator of the Wnt signaling pathway to LRP receptors or frizzled receptors activates Dvl and suppresses GSK-3 expression, which leads to an accumulation of β-catenin in the cytoplasm. The high levels of β-catenin then migrate to the nucleus and bind to LEF-1/TCF, thereby reducing the expression levels of PPARγ and C/EBPα. A substantiating study highlighted that activated Dvl represses the activity of the destruction complex (GSK-3, APC, Axin, β-TrCP/Slimb) and stabilizes β-catenin by increased phosphorylation [35]. In addition, Wnt1 is also known as an inducible signaling pathway protein 2 (WISP2), and as a suppressor adipokine for adipogenic commitment to form the cytosolic complex with PPARγ and Zfp423, which is separated by BMP4 by interaction with Smad [36], while Zfp423 translocates to the nucleus and activates PPARγ, ultimately committing cell differentiation toward the adipose lineage.

Identification of increased levels of SFRP5 expression in the fat tissue of obese mice and humans have been reported. The expression of SFRP5 is slowly induced in the process of differentiation of white and brown adipocytes and highly elevated in mature adipocytes [5]. A previous study demonstrated in their SFRP5-deficient mouse models that inducing severe macrophage infiltration could cause serious glucose intolerance, insulin resistance, as well as inflamed adipose tissue [30]. Multiple studies have demonstrated the regulatory roles of SFRPs as extracellular Wnt antagonists in various processes during adipogenesis [37,38]. The association of Wnt signaling with adipogenesis was identified based on evidence provided, suggesting that the expression levels of Wnt10b were significantly reduced during lipid metabolism [39]. Wnt10b inhibits adipocyte differentiation by decreasing the levels of adipogenic transcription factors such as PPARγ and C/EBPα [40]. The Wnt signaling produced as a result of Wnt10b mediates the balance amongst adipogenic, osteoblastogenic, and myogenic conditions, resulting in decreased adipogenesis [41,42]. A positive feedback system is formed, whereby SFRP5 expression suppresses the Wnt signaling. It is speculated that the mechanism for this feedback system involves the neutralization of Wn5a by SFRP5. This phenomenon has been seen to be present during obesity whereby the stimulation of adipose tissue results in the activation of JNK1, and leads to the onset of metabolic disorders and macrophage activation. However, an element of controversy exists amongst different studies regarding the effects of SFRP5 on obesity. Lower SFRP5 levels have been detected in obese subjects in contrast with lean subjects, with studies drawing correlations between SFRP5 and adiposity indicators such as body mass index (BMI), waist-hip ratio (WHR), body fat percentage, and lipid profile [43]. Schulte et al. [44] pointed out that SFRP5 expression, unlike Wnt5a, does vary significantly between obese and lean subjects based on ELISA. However, Ouchi et al. [15] emphasized the transient role of SFRP5 in his study, suggesting that SFRP5 expression is increased in adipose tissue but reduced in response to serious obesity-related metabolic dysfunctions. This ultimately demonstrates that the up-regulation of SFRP5 in obese and diabetic mice alleviates metabolic disorders and suppresses adipose tissue inflammation [15]. These discrepancies may however be accounted for by the different diets the animals were placed on in the studies, differences in age of the animals at the time of detection, and measurements conducted. Based on the observations and analysis of the model of adipogenesis by Mori et al. [45], SFRP5 promotes adipocyte growth by repressing Wnt signaling and decreasing oxidative metabolism as an endogenous suppressor of adipogenesis. This current review holds the stance that SFRP5 promotes adipocyte differentiation by blocking the Wnt signaling pathway. Our review has been supported by findings conducted by Rulifson et al. [46] and Van Camp et al. [47], which provided distinct evidence indicating that a significant increase in SFRP5 expression was observed amongst adipose tissues in obese mice and that genetic variation in SFRP5 could determine the distribution and volume of both subcutaneous and abdominal fat in obese males, respectively.

## SFRP5 and inflammation

SFRP5 accelerates adipocyte differentiation and lipid storage in adipocytes by repressing the Wnt signaling, thereby further attenuating inflammation [47]. SFRP5 is highly expressed in white adipocytes, and has been noted to suppress the binding of Wnt proteins to its receptors. Such an example is the suppression of Wnt5a binding to its receptor which shares a negative correlation with the onset of inflammatory responses [48]. When the expression of SFRP5 is reduced, the expression of Wnt5a, an antagonizing target of SFRP5, is elevated in white adipose tissues of obese rodents and humans. This suggests that there may be a potential role of SFRP5 in inhibiting the inflammatory effects of Wnt5a in adipose tissues [49]. Additionally, SFRP5 displays a substantially higher protein expression level in white adipose tissues compared with other tissues [50]. The positive correlation between SFRP5 with markers of oxidative stress and insulin resistance has been highlighted amongst predominantly overweight and obese Caucasian populations, suggesting that the action of SFRP5 in humans may be affected by metabolic and inflammatory conditions [25]. Rulifson et al. [46] reported that in response to metabolic stress, SFRP5 production is up-regulated in adipocytes and exerts its anti-inflammatory effects through regulating the JNK signaling pathway to improve insulin resistance.

As an anti-inflammatory adipokine, SFRP5 could be negatively modulated in the development of obesity, contributing to an unfavorable metabolic phenotype [29]. Accordingly, Catalán et al. [48] revealed that activated Wnt signaling via the increased expression of Wnt5A and decreased expressions of SFRP5 may lead to a pro-inflammatory state in visceral adipose tissue, promoting the progression of obesity-associated comorbidities. Moreover, SFRP5 sequesters and binds to Wnt5a in the extracellular space of adipose tissues in order to block the activation of JNK1, which reduces Wnt signaling, thereby attenuating chronic inflammatory conditions, which ultimately improves insulin sensitivity [29]. Amongst obese individuals, it is critical that β cells in the pancreas need to be maintained in normal ranges in order to sustain normal glucose homeostasis. There have been reports suggesting that inflammatory cytokines play a pivotal role in β-cell dysfunction, which can be altered as a consequence of dysfunctional adipocyte in obesity [51]. Studies have indicated that the Wnt signaling pathway may be a possible regulator in the process of β-cell proliferation and β-cell mass expansion. A study demonstrated that amongst cafeteria-diet-fed rat models and obese human beings, decreased expression of SFRP5 in the pancreatic islets was observed [52]. The specific knockdown of SFRP5 has been found to produce an increase in β-cell proliferation, via activation of the Wnt signaling pathway. In addition, IGF binding protein 3, which is generated from visceral adipose tissues, could mediate SFRP5 expression in β cells. Since the serum SFRP5 concentration is negatively correlated with IL-1β, tumor necrosis factor-α (TNF-α), cholesterol, and apoprotein B levels, Wnt5 is inversely correlated with adiposity and directly associated with responses to insulin. Better knowledge of the effect of Wnt signaling in obese individuals, insulin resistance, and inflammation remains an absolute necessity for the prevention and treatment of metabolic syndrome, diabetes, and other cardiovascular diseases [53]. Nakamura et al. [17] provided convincing evidence supporting the hypothesis that serum SFRP5 levels in patients with coronary artery disease (CAD) are significantly reduced, suggesting that a negative correlation may exist between the severity of CAD and serum levels of SFRP5. Thus, determining the levels of serum SFRP5 may be a useful indicator in aiding the diagnosis and severity of CAD in patients [54]. In bone marrow-derived macrophages, the forced expression of SFRP5 was shown to inhibit the effects of Wnt5a on its facilitation of JNK activation in addition to increasing cardiac inflammatory gene expression. This suggests that SFRP5 plays a key role in antagonizing inflammatory responses following situations of ischemia and reperfusion in the heart, possibly through a mechanism involving Wnt5a/JNK signaling [17].

## SFRP5 and insulin resistance in T2DM

T2DM is significantly influenced by chronic low-grade inflammation as a response stimulated by metabolic stress [51]. In response to obesity and T2DM, it was found that the plasma levels of SFRP5 were significantly lower in Chinese obese individuals as well as T2DM subjects compared with normal subjects, in which SFRP5 was an independent factor influencing glucolipid metabolism, inflammation, and IR. This highlights the crucial role of SFRP5 in the pathogenesis of obesity and T2DM [55]. SFRP5 was identified by Cheng et al. [56] as a protective factor in the pathogenesis of autoimmune diabetes, suggesting it could facilitate a novel aspect of the treatment of patients with T2DM as well as latent autoimmune diabetes in adults. Furthermore, plasma SFRP5 has been linked to homeostasis model assessment of insulin resistance (HOMA-IR), uric acid, diabetes duration, and BMI. Particularly, the correlation between plasma SFRP5 and HOMA-IR still exists after adjustment for diabetes duration and BMI [56]. On the contrary, a study conducted by Canivell et al. [57] demonstrated that serum SFRP5 levels were increased in recently diagnosed drug-naive T2DM patients when compared with prediabetic subjects and normal glucose tolerance states. This finding was supported by a study that identified that low serum levels of SFRP5 played a contributory factor in the pathophysiology of obesity and T2DM, while suggesting that HOMA-IR, BMI, and triglycerides were independent, related factors capable of influencing the plasma levels of SFRP5 [58]. Furthermore, a multiple linear regression analysis revealed that only fasting glucose levels were positively associated with plasma SFRP5 levels [16]. The protective role of SFRP5 in the development of T2DM has been determined by findings that showed increased insulin sensitivity, decreased macrophage infiltration, and pro-inflammatory protein production [59]. The expression and transcription of SFRP5 have been reported to share a negative association with insulin resistance [23]. A reduction in SFRP5 transcription could result in an increase in the ratio of Wnt5a to SFRP5, which is correlated with inhibited glucose and insulin signaling [44]. Components of the Wnt pathway such as the Wnt co-receptors LRP5/6, a Wnt ligand, and major Wnt effector, TCF7L2 have been shown to be involved in glucose and lipid metabolism and consequently affect the development of T2DM and other metabolic diseases [60,61].

SFRPs share an antagonistic relationship with Wnt signaling through binding to Wnts or their receptors in the plasma membrane [62]. SFRP5 is able to compete with Wnt5a to activate the Wnt signaling by binding to the frizzled receptors [8], followed by the activation of the Wnt signaling pathway by Wnt5a, stimulating the phosphorylation of JNK [11]. JNK activation or overexpression promotes serine phosphorylation of IRS-1, which is a transcriptional target of Wnt, and suppresses its tyrosine phosphorylation (for insulin stimulation), ultimately resulting in increased insulin resistance [63,64]. Up-regulation of SFRP5 inhibits insulin resistance and inflammation by activating JNK mediated by Wnt in adipocytes and macrophages, thereby providing systemic effects [65]. Yoon et al. [66] indicated that the increased levels of IRS-1, a transcriptional target of Wnt, facilitate the activation of mitochondrial biogenesis, leading to DNA damage and accelerated cellular senescence in primary cells. Furthermore, up-regulated IRS-1 enhances insulin signaling, promoting the potential use of how Wnt proteins may be used to regulate glucose homeostasis in insulin-responsive cell types [66]. Moreover, Abiola et al. [67] suggested that the activation of Wnt/β-catenin signaling in skeletal muscle cells resulted in improved insulin sensitivity according to the following three mechanisms: decreasing intramyocellular lipid deposition via the down-regulation of SREBP-1c; increasing the effects of insulin through the differential activation of the Akt/PKB and AMPK pathways; and inhibiting the activation of the MAPK pathway.

**Figure 1:**
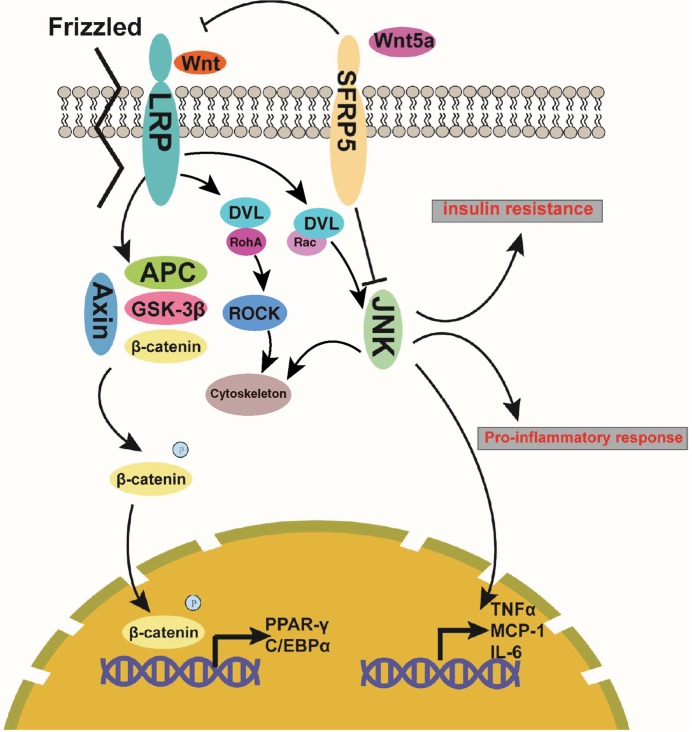
The mechanistic map of action of SFRP5 in adipogenesis through the Wnt/β-catenin signaling pathway and signaling map in relation to the effect of SFRP5 on inflammation and insulin sensitivity Frizzled receptors can form complex with LRP in a manner of transmembrane protein and then the extracellular Wnt protein is combined with the complex, making the signal transmitted into cells. Following this, Axin/APC/GSK-3β complex is formed and combined with β-catenin, which leads to the activation of β-catenin through phosphorylation. Activation of β-catenin (transferring from cytoplasm to nucleus) thereby commits to the transcription of the target genes in the downstream, such as PPAR-γ and CEBPα. For the Wnt/PCP signaling pathway, Wnt binds to Frizzled receptors on the surface of cells and subsequently delivers signals into the cells to activate the downstream GTPase Rho and JNK, thus participating in the cytoskeleton and regulation of downstream genes of JNK signaling. SFRPS is capable of preventing the binding of Wnt and Frizzled proteins through its combination with Wnt protein, thus blockading the Wnt signaling, inhibiting the phosphorylation activation of β-catenin, and the transcription of the target genes (PPAR-γ and CEBPα) in the downstream. In addition, SFRPS has the ability to repress the JNK signaling via its combination with Wnt5a, leading to the suppression of TNF-α, MCP-1, and IL-6; as a result, the pro-inflammatory response and insulin resistance are reduced. Abbreviation: IL-6, interleukin-6.

A majority of studies supported the beneficial action of SFRP5 in glucose tolerance [55]. However, a report conducted by Rulifson et al. [46] provided contradictory findings that overexpressed SFRP5 actually reduced glucose intolerance and produced hyperglycemia, while the treatment of monoclonal antibody against SFRP5 intensified glucose metabolism. Studies have shown that SFRP5 expression is suppressed by glucose through the PI3K/AKT signaling pathway, with promoted viability of rat pancreatic β cells [68]. Circulating SFRP5 expression during an oral glucose tolerance test was both rapidly and markedly reduced in response to hyperglycemia [47]. Additionally, in the pancreatic islets of rats placed on a cafeteria diet, SFRP5 levels were found to be significantly reduced which is implicated in β-cell viability during the β-cell mass expansion in obesity via the Wnt signaling pathway [52]. Other SFRP members such as SFRP4, have been reported to play a role in insulin sensitivity, whereby its increased production has led to reduced glucose tolerance. This was achieved by reducing the islet expression levels of calcium channels and repressing insulin exocytosis, which suggests that SFRP4 may form a link between reduced insulin secretion and islet inflammation [69]. It has previously been suggested that SFRP5 is a protective adipokine in hepatic steatosis, glucose intolerance and fibrosis [70]. SFRP5 administration in diabetic and obese mice leads to decreased glucose intolerance, brought about by high-caloric intake and characterized by macrophage infiltration and hepatic steatosis in adipose tissues [25]. Taken together, we propose that SFRP5 binds to Wnt5a and blocks the Wnt signaling pathway, thereby reducing chronic inflammation and improving insulin sensitivity. The mechanistic map for the action of SFRP5 in lipid metabolism, inflammation, and insulin sensitivity through the Wnt signaling pathway as illustrated in Figure 1.

## Challenges, unknowns, and therapeutic opportunities

In this review, we have speculated that the activation of the Wnt signaling pathway can facilitate chronic low-grade inflammation in adipose tissue. Despite the ongoing and available research, there is a need for further investigation to determine whether the Wnt signaling pathway can exclusively directly affect adipose tissue inflammation in obese individuals or physiological functions. However, the relationship between SFRP5 and the Wnt signaling pathway transduction in the liver and skeletal muscles as well as the insulin signaling network also requires further exploration. Do the intravenous injections of SFRP5 directly block the Wnt signaling pathway in the liver and skeletal muscles or do they indirectly enhance the insulin sensitivity? Further elucidation into whether SFRP5 levels are regulated during the development and progression of obesity and diabetes mellitus is required, since there remain elements of controversy as to whether they increase all the time or fall below at particular point, thereby influencing metabolism. In conclusion, this review speculates the role of SFRP5 in the pathogenesis of lipid metabolism, inflammation, and T2DM. Further understanding of the biological functions implicated may pave the way for SFRP5 to serve as a promising novel therapeutic target in the treatment of obesity, T2DM, and other metabolic diseases.

## Abbreviations

APC: adenomatous polyposis coli
AMPK: AMP-activated protein kinase
BMI: body mass index
BMP4: bone morphogenetic protein 4
CAD: coronary artery disease
CCND1: cyclin D1 gene
C-EBP: CCAAT/enhancer binding protein
HOMA-IR: homeostasis model assessment of insulin resistance
IRS-1: insulin receptor substrate-1
JNK: c-Jun N-terminal kinase
LEF-1/TCF: lymphoid enhancer-binding factor 1/T cell-specific transcription factor
LRP: low-density lipoprotein receptor-related protein
MCP-1: monocyte chemoattractant protein-1
MMP-7: matrix metalloproteinase-7
PCP: planar cell polarity
PI3K: phosphatidylinositol-3-kinase
PKB: protein kinase B
PPARγ: peroxisome proliferator-activated receptor γ
SFRP: secreted frizzled-related protein
T2DM: type 2 diabetes mellitus
Zfp423: zinc finger protein 423
